# Supplementary material for: Antibodies to the RBD of SARS-CoV-2 spike mediate productive infection of primary human macrophages
Source: Nat Commun. 2024 Dec 30;15:10764. doi: 10.1038/s41467-024-54458-w (PMC11686093; doi:10.1038/s41467-024-54458-w)
Supplement: Supplementary file 1 — Supplementary Information [file 41467_2024_54458_MOESM1_ESM.pdf]

Supplementary Figure 1

Dual HeLa-ACE2/THP-1 assay set-up with SARS-CoV-1 and additional information relating to Figures 1 and 2

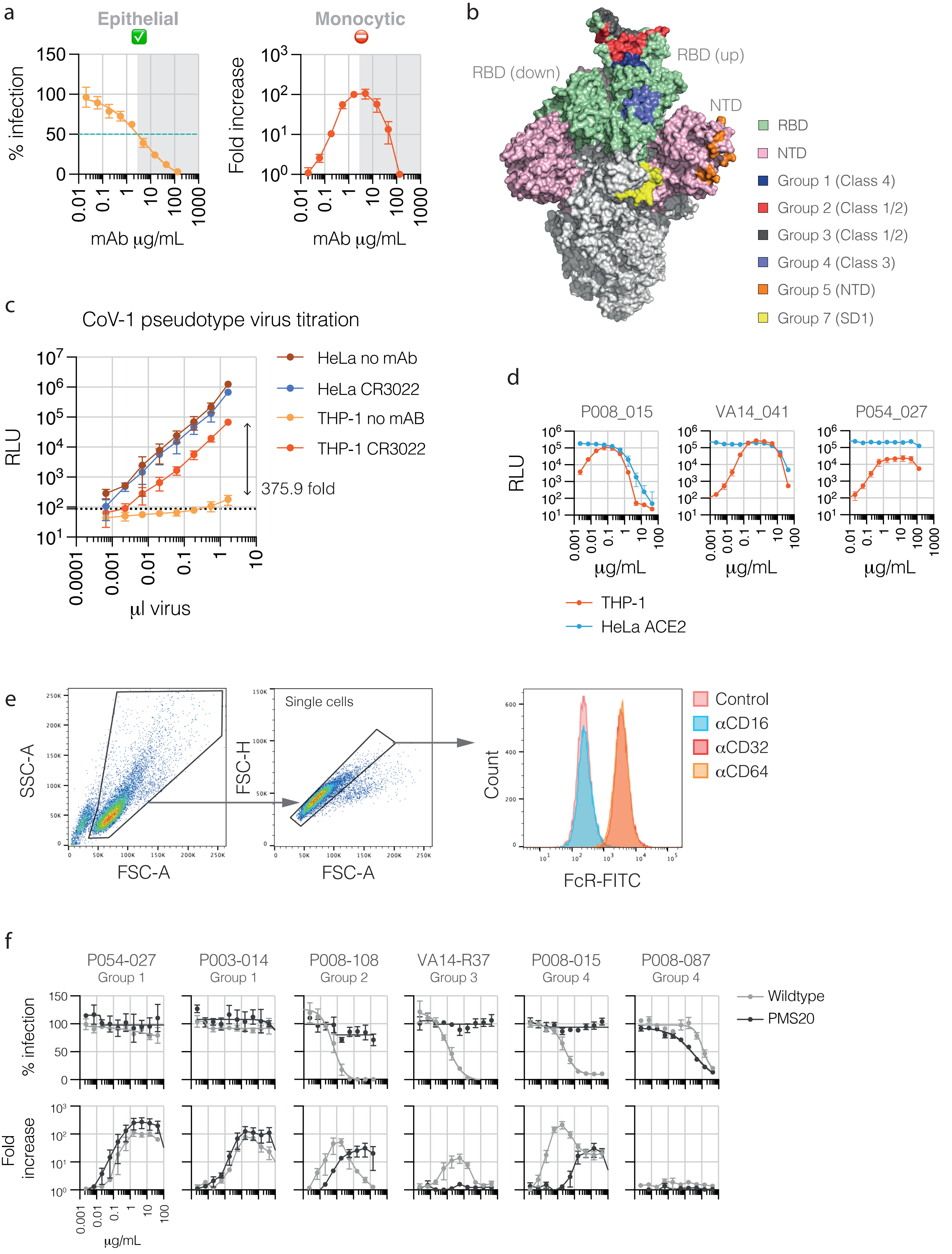

Supplementary Figure 1

Dual HeLa-ACE2/THP-1 assay set-up with SARS-CoV-1 and additional information relating to figures 1 and 2

(a) Assays were performed in parallel on permissive epithelial cells (HeLa-ACE2) and non-permissive monocytic cells (THP-1) using HIV-1-based pseudoviruses bearing SARS-CoV-1 spikes pre-incubated with 3-fold dilutions of anti-SARS-CoV-1 mAb CR3022. Infection was measured by luciferase assay 48 hours later, with % infection and fold increase calculated relative to infection levels in the absence of antibody (in the case of THP-1 cells this is background relative light units (RLU)). Grey shaded areas indicate mAb concentrations at which greater than 50% neutralisation occurs. Means are derived from n=3 independent experiments, error bars ±SEM.

(b) Surface representation of SARS-CoV-2 spike showing antibody binding epitopes as characterised previously (PDB: 6Xm0) [1, 43] and with corresponding antibody binding classes as characterised by Barnes et al. [2] shown in parentheses.

(c) Increasing quantities of HIV-1-based pseudoviruses with SARS-CoV-1 spikes were incubated with CR3022 (2 μg/ml) or no mAb as a control for 1 hour, before adding to permissive HeLa-ACE2 cells or non-permissive THP-1 cells. 48 hours later raw RLUs were determined by luciferase assay. Means are derived from n=3 independent experiments, error bars ±SD.

(d) Raw RLU values from three mAb titrations shown in Figure 1a. Means are derived from n=2 independent experiments, error bars ±SEM.

(e) Flow cytometry gating strategy and histograms showing major Fc receptors CD64 (FcγRI), CD32 (FcγRII) and CD16 (FcγRIII) expression on THP-1 cells.

(f) Representative mAbs from each of the four RBD binding groups shown in (b) were tested in parallel HeLa-ACE2 and THP-1 assays, as for Figure 2, using pseudoviruses with PMS20 spikes – a synthetic spike with maximum wave 1 polyclonal neutralisation resistance – or its unmutated wildtype equivalent [46]. Means are derived from n=3 independent experiments, error bars ±SEM.

Supplementary Figure 2

Infection of differentiated THP-1 cells and iPSC-derived macrophages by SARS-CoV-2

a

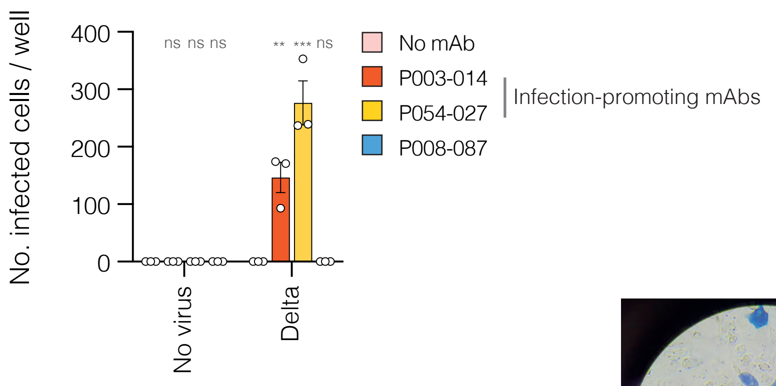

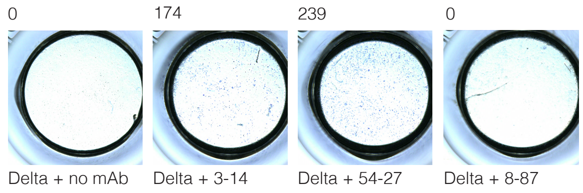

Well overviews - blue staining = SARS-CoV-2 nucleocapsid.

Numbers above images show infected cell count.

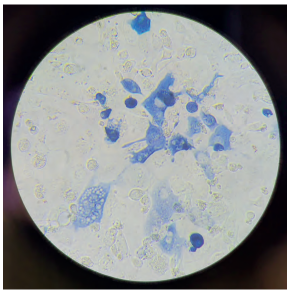

Zoomed-in field of view, showing foci of infection, including cytoplasmic staining and fused cells

b

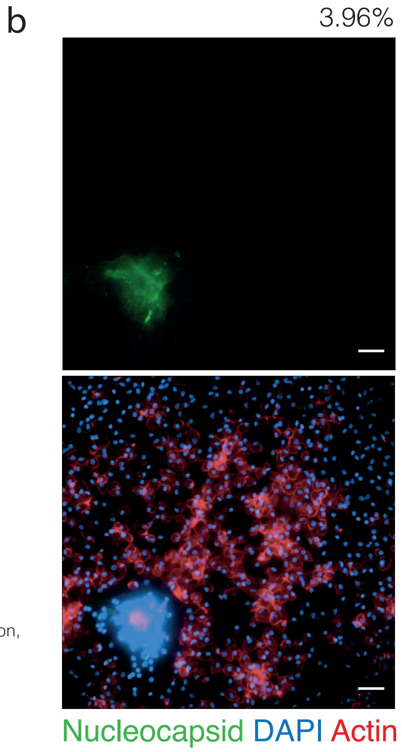

c

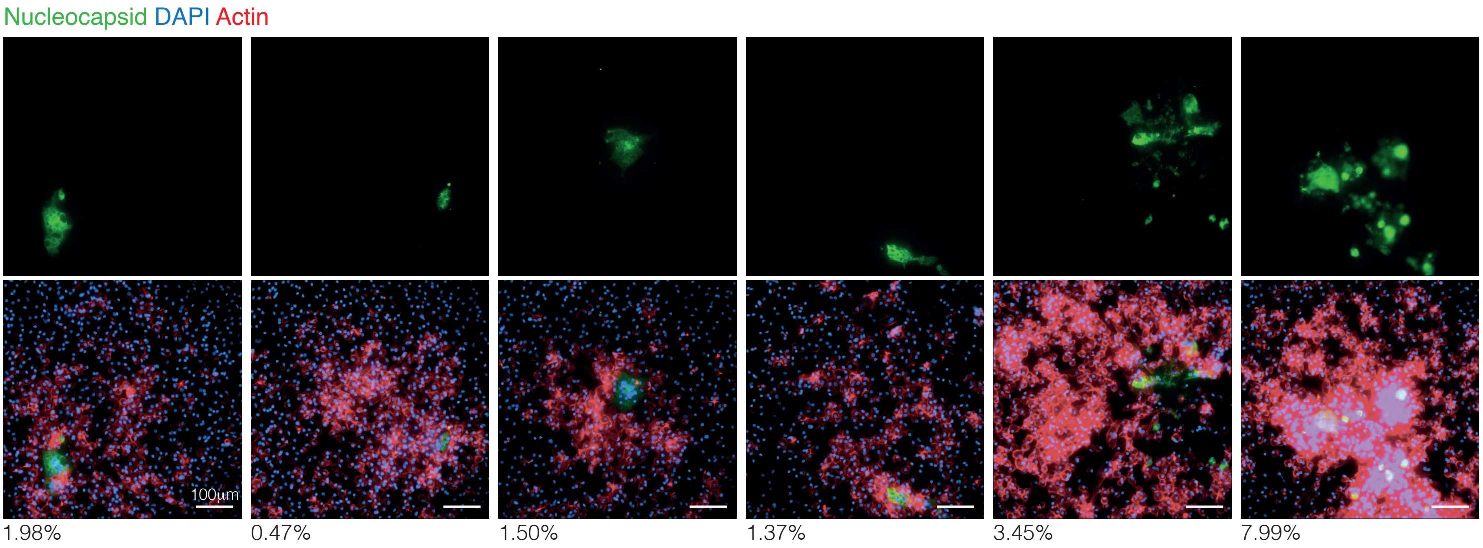

Supplementary Figure 2

Infection of differentiated THP-1 cells and iPSC-derived macrophages by SARS-CoV-2

(a) Delta virus was preincubated with infection-promoting mAbs P003_014, P054_027 or non infection-promoting control mAb P008_087 at 6 μg/ml prior to addition to PMA-differentiated THP-1 cells at an MOI of 1. 72 hours later cells were intracellularly stained for SARS-CoV-2 nucleocapsid and the number of infected cells per well determined by ELISPOT. Means are derived from n=3 independent experiments, error bars ±SEM. Infection levels in the presence of each mAb were compared to no mAb using two-way ANOVA with mixed effects analyses. P= 0.0039 for mAb P003_014 and 0.0001 for P054_027 (>0.1 (ns), <0.1 (*), <0.01 (**), <0.001 (***), <0.0001 (****)).

(b) Representative images from XEGX_1 iPSC-derived macrophage infections shown in Figure 5a. Green, SARS-CoV-2 nucleocapsid; red, actin; blue, DAPI. Scale bar 50 μm. % infection for the field of view shown is indicated above the image.

(c) Additional representative images of iPSC-derived macrophages infected with SARS-CoV-2, from experiments shown in Figure 5a. Green, SARS-CoV-2 nucleocapsid; red, actin; blue, DAPI. Scale bars 100 μm. Top row show nucleocapsid staining only. % infection for each individual field of view is shown below each image.

Supplementary Figure 3

Additional images of SARS-CoV-2 infected macrophages, Fc receptor expression on primary human macrophages and treatment with inhibitors

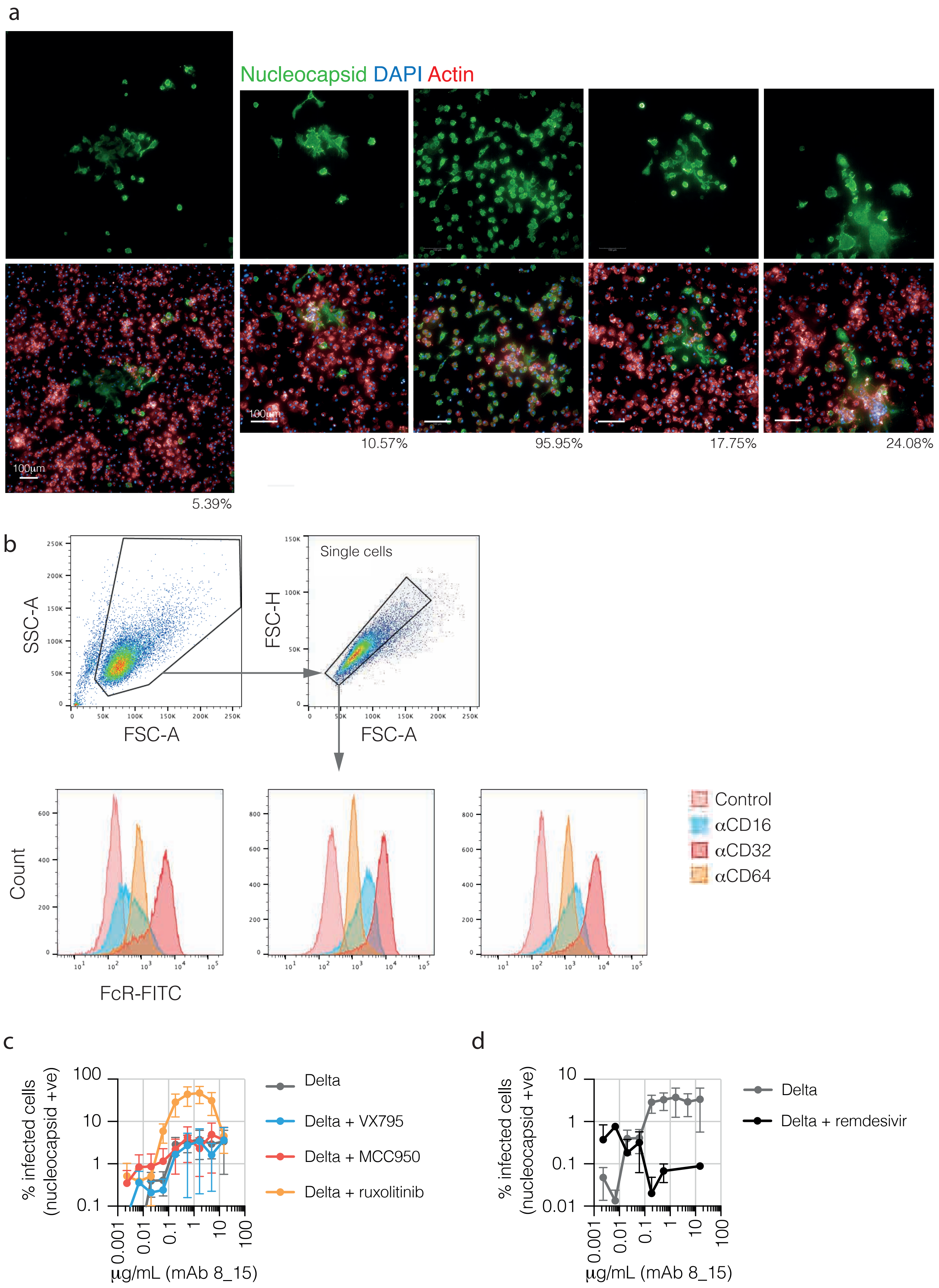

Supplementary Figure 3

Additional images of SARS-CoV-2 infected macrophages, Fc receptor expression on primary human macrophages and treatment with inhibitors

(a) Additional images of primary human macrophages infected with SARS-CoV-2, from experiments shown in Figure 5b and 6a. Scale bars 100 μm. Green, SARS-CoV-2 nucleocapsid; red, actin; blue, DAPI. % infection for each individual field of view is shown below each image.

(b) Flow cytometry gating strategy and histograms showing the expression of major Fc receptors CD64 (FcγRI), CD32 (FcγRII) and CD16 (FcγRIII) on primary human monocyte-derived macrophages. A representative example of the gating strategy is shown for one donor, and Fc receptor expression is shown for three individual donors.

(c-d) Primary monocyte-derived macrophages were infected as in Figure 6a with delta virus at an MOI of 1, pre-incubated with mAb P008_015 in the presence of caspase-1 and -2 inhibitor VX-755, NLRP3 inflammasome inhibitor MCC950 or ruxolitinib (c) or remdesivir (d). Means are derived from n=3 independent experiments, error bars ±SEM.

Supplementary Figure 4

Correlations between productive infection and cytokine production in primary human macrophages

a

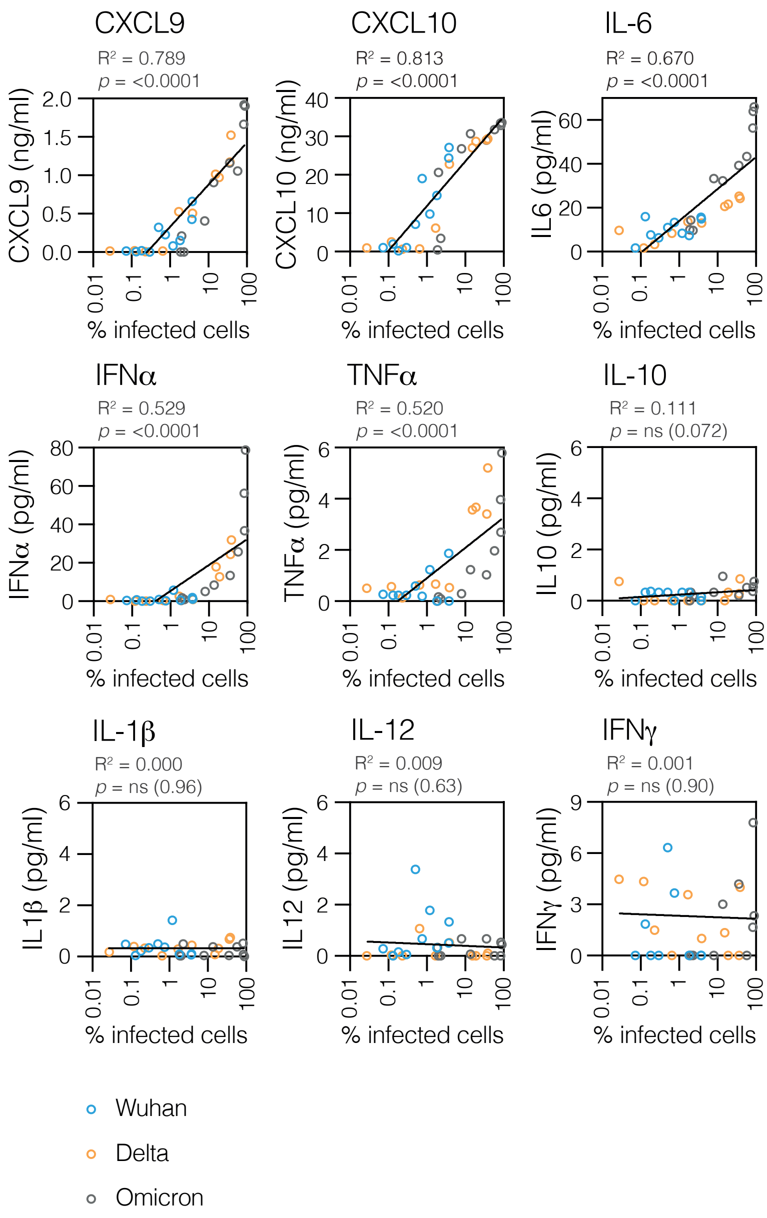

b

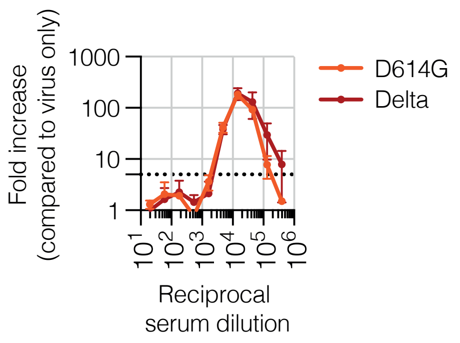

Supplementary Figure 4

Correlations between infection and cytokine production in primary human macrophages

(a) Correlations between % infected cells in the presence of ruxolitinib and production of cytokines for experiments shown in Figure 6a. R^2 and *p* values were determined by two-tailed Pearson correlation, >0.1 (ns), <0.1 (*), <0.01 (**), <0.001 (***), <0.0001 (****).

(b) Serum sample from a delta-infected individual, shown in Figure 7b, was tested in THP-1 assays with delta and D614G spike-pseudotypes. Means are derived from n=3 independent experiments, error bars ±SEM.

Analysis Sequence "20230123 SP Primary Macs Thr@12000"

Input Image	Input
	**Flatfield Correction :** Basic<br>Brightfield Correction<br>**Stack Processing :** Individual Planes<br>**Min. Global Binning :** Dynamic

Find Nuclei	Input	Method	Output
	**Channel :** DAPI<br>**ROI :** None	**Method :** C<br>Common Threshold : 0.4<br>Area : > 30 µm²<br>Splitting Coefficient : 7<br>Individual Threshold : 0.4<br>Contrast : > 0.1	Output Population : Nuclei

Find Cytoplasm	Input	Method	Output
	**Channel :** Alexa 647<br>**Nuclei :** Nuclei	**Method :** C<br>Common Threshold : 0.2<br>Individual Threshold : 0.3	

Select Population	Input	Method	Output
	**Population :** Nuclei	**Method :** Common Filters<br>Remove Border Objects<br>Region : Cell	Output Population : Nuclei Selected

Calculate Morphology Properties (2)	Input	Method	Output
	**Population :** Nuclei Selected<br>**Region :** Cell	**Method :** Standard<br>Area<br>Roundness<br>Width<br>Length<br>Ratio Width to Length	Property Prefix : Cell

Calculate Intensity Properties	Input	Method	Output
	**Channel :** DAPI<br>**Population :** Nuclei	**Method :** Standard<br>Mean	Property Prefix : Intensity Cell DAPI

	**Region :** Cell	Standard Deviation<br>Coefficient of Variance<br>Median<br>Sum<br>Maximum<br>Minimum	

Calculate Intensity Properties (2)	Input	Method	Output
	**Channel :** Alexa 488<br>**Population :** Nuclei Selected<br>**Region :** Cell	**Method :** Standard Mean	Property Prefix : Intensity Cell Alexa 488

Select Population (2)	Input	Method	Output
	**Population :** Nuclei Selected	**Method :** Filter by Property<br>Intensity Cell Alexa 488 Mean : > 12000	Output Population : GFP+

Define Results	Results
	**Method :** List of Outputs<br>**Population : Nuclei Selected**<br>Number of Objects<br>Apply to All : Mean+StdDev<br>Cell Area [μm²] : Mean+StdDev<br>Cell Roundness : Mean+StdDev<br>Cell Width [μm] : Mean+StdDev<br>Cell Length [μm] : Mean+StdDev<br>Cell Ratio Width to Length : Mean+StdDev<br>Intensity Cell Alexa 488 Mean : Mean+StdDev<br>GFP+ : Mean+StdDev<br><br>**Population : Nuclei**<br>Apply to All : None<br><br>**Population : GFP+**<br>Number of Objects<br>Apply to All : Mean+StdDev<br>Cell Area [μm²] : Mean+StdDev<br>Cell Roundness : Mean+StdDev<br>Cell Width [μm] : Mean+StdDev<br>Cell Length [μm] : Mean+StdDev<br>Cell Ratio Width to Length : Mean+StdDev<br>Intensity Cell Alexa 488 Mean : Mean+StdDev<br><br>**Method :** Formula Output<br>Formula : 100*a/b<br>Population Type : Objects<br>Variable a : GFP+ - Number of Objects

Variable b : Nuclei Selected - Number of Objects
Output Name : % Cells +

Object Results
Population : Nuclei Selected : None
Population : Nuclei : None
Population : GFP+ : None

Acapella version: 5.1.2.129056. Timestamp: 2023-09-05 09:52:44 -0400.

Supplementary Figure 5

Analysis pipeline for high-content imaging.

Supplementary Table 1
Baseline characteristics of the 43 human serum sample donors (SARS-CoV-2 infected individuals).

**Sex**	
Male, no. (%)	24 (55.8)
Female, no. (%)	19 (44.2)
**Age**	
Mean (range)	48.8 (17-91) years
**Severity**	
0	7
1	10
2	9
3	1
4	11
5	5

Supplementary Table 2
Baseline characteristics of human blood donors used for monocyte/macrophage isolation.

**Sex**	
Male, no. (%)	7 (77.8)
Female, no. (%)	2 (22.2)
**Age**	
Mean (range)	27 (24-31)
**Severity**	
No underlying health conditions, no. (%)	9 (100)

Supplementary Table 3
Analytes measured by CBA with BD Biosciences reference numbers.

Analyte	BD Biosciences ref. no.
IL-1β	558279
IL-6	558276
IL-10	558274
IL-23/IL-12p40	560154
TNFα	560112
IFNα	560379
IFNɣ	558269
CXCL9 (MIG)	558286
CXCL10 (IP-10)	558280
