# Supplementary material for: Antibodies to the RBD of SARS-CoV-2 spike mediate productive infection of primary human macrophages
Source: Nat Commun. 2024 Dec 30;15:10764. doi: 10.1038/s41467-024-54458-w (PMC11686093; doi:10.1038/s41467-024-54458-w)
Supplement: Supplementary file 2 — Reporting Summary [file 41467_2024_54458_MOESM2_ESM.pdf]

Corresponding author(s): Suzanne Pickering
Last updated by author(s): 2024/09/30

Reporting Summary

Nature Portfolio wishes to improve the reproducibility of the work that we publish. This form provides structure for consistency and transparency in reporting. For further information on Nature Portfolio policies, see our Editorial Policies and the Editorial Policy Checklist.

Statistics

For all statistical analyses, confirm that the following items are present in the figure legend, table legend, main text, or Methods section.

n/a	Confirmed	
☐	☒	The exact sample size (*n*) for each experimental group/condition, given as a discrete number and unit of measurement
☐	☒	A statement on whether measurements were taken from distinct samples or whether the same sample was measured repeatedly
☐	☒	The statistical test(s) used AND whether they are one- or two-sided<br>*Only common tests should be described solely by name; describe more complex techniques in the Methods section.*
☒	☐	A description of all covariates tested
☐	☒	A description of any assumptions or corrections, such as tests of normality and adjustment for multiple comparisons
☐	☒	A full description of the statistical parameters including central tendency (e.g. means) or other basic estimates (e.g. regression coefficient) AND variation (e.g. standard deviation) or associated estimates of uncertainty (e.g. confidence intervals)
☐	☒	For null hypothesis testing, the test statistic (e.g. *F*, *t*, *r*) with confidence intervals, effect sizes, degrees of freedom and *P* value noted<br>*Give P values as exact values whenever suitable.*
☒	☐	For Bayesian analysis, information on the choice of priors and Markov chain Monte Carlo settings
☒	☐	For hierarchical and complex designs, identification of the appropriate level for tests and full reporting of outcomes
☒	☐	Estimates of effect sizes (e.g. Cohen's *d*, Pearson's *r*), indicating how they were calculated

Our web collection on statistics for biologists contains articles on many of the points above.

Software and code

Policy information about availability of computer code

Data collection	Harmony 4.9 software was used to acquire images on the Operetta CLS High Content Analysis System.<br>BD FACSDiva Software v9.2 was used to acquire data on the FACSCanto II.<br>AID EliSpot 8.0 software was used to count plaques for differentiated THP-1 infection assays.
Data analysis	FlowJo v10.8.1 (BD) was used for flow cytometry analysis.<br>Harmony 4.9 software was used to analyse images acquired by high content imaging.<br>Graphpad Prism v10 was used for data analysis.

For manuscripts utilizing custom algorithms or software that are central to the research but not yet described in published literature, software must be made available to editors and reviewers. We strongly encourage code deposition in a community repository (e.g. GitHub). See the Nature Portfolio guidelines for submitting code & software for further information.

Data

Policy information about availability of data

All manuscripts must include a data availability statement. This statement should provide the following information, where applicable:

- Accession codes, unique identifiers, or web links for publicly available datasets
- A description of any restrictions on data availability
- For clinical datasets or third party data, please ensure that the statement adheres to our policy

Datasets generated and/or analysed during the current study are included in the paper or are appended as supplementary data. Source data are provided with this paper.

Research involving human participants, their data, or biological material

Policy information about studies with human participants or human data. See also policy information about sex, gender (identity/presentation), and sexual orientation and race, ethnicity and racism.

Reporting on sex and gender	Primary PBMC were isolated from anonymous healthy donors at KCL as a source of monocyte-derived macrophages for discovery experimentation. Although both male and female donors were included, this study sought to investigate whether such cells could become infected, not to examine relative permissivity effects of sex or gender and so were not formally balanced. Three individual donors were used per experiment for biological replicates and were subject to availability of donors on the day of blood draw, therefore it was not possible or appropriate to balance sex or gender in this instance. For the serum samples from SARS-CoV-2-infected individuals, samples were scavenged before discard, following collection as part of routine clinical care, as covered by ethics granted by South Central Hampshire B REC (20/SC/0310). This was an observational study based on the availability of pre-existing samples, therefore, written informed consent was not required and we as researchers had no control over the sex/gender distribution.
Reporting on race, ethnicity, or other socially relevant groupings	N/A
Population characteristics	N/A
Recruitment	For serum samples from SARS-CoV-2-infected individuals, no participants were enrolled as all samples pre-existed.<br>For blood samples obtained as a source of CD14+ monocytes, healthy donors were recruited from volunteers from King's College London. All volunteers gave written informed consent prior to participation.
Ethics oversight	Ethical approval to draw blood from healthy donors as a source for primary CD14+ monocytes was granted by the King's College London Infectious Disease BioBank Local Research Ethics Committee – approvals SN1-100818 and SN1-160322. For the serum samples from SARS-CoV-2-infected individuals, surplus serum samples taken as part of routine clinical care were retrieved at the point of being discarded and as described previously in Seow, J., et al. Nat Microbiol, 2020. 5(12): p. 1598-1607., with the ethical oversight for this study being the same as the original study and approved by South Central Hampshire B REC (20/SC/0310). The collection of surplus or discarded samples and linked-anonymised clinical information bypassed the requirement for written consent.

Note that full information on the approval of the study protocol must also be provided in the manuscript.

Field-specific reporting

Please select the one below that is the best fit for your research. If you are not sure, read the appropriate sections before making your selection.

[x] Life sciences [ ] Behavioural & social sciences [ ] Ecological, evolutionary & environmental sciences

For a reference copy of the document with all sections, see nature.com/documents/nr-reporting-summary-flat.pdf

Life sciences study design

All studies must disclose on these points even when the disclosure is negative.

Sample size	The number of serum samples from individuals with SARS-CoV-2 delta infection was determined by the availability of pre-existing acute samples (between 0-21 days post onset of symptoms). Experiments conducted in primary monocyte-derived macrophages were performed on cells from at least three different donors as is standard protocol for biological replicates.
Data exclusions	Serum samples from individuals with SARS-CoV-2 delta infections were excluded if the individuals had undergone monoclonal antibody therapy.
Replication	The ability of defined mAbs to mediate infection of monocytes/macrophages by infectious SARS-CoV-2 was first optimised on THP-1 cells lines, then subsequently confirmed on differentiated THP-1 cells and iPSC-derived macrophages from three different genotypes (i.e. biological triplicates). These results were then reproduced successfully on primary human monocyte-derived macrophages from at least three individual donors. All means and error bars were derived from biological rather than technical replicates. All attempts at replication were successful.

Randomization	Randomization was not necessary because studies conducted on serum samples from SARS-CoV-2-infected individuals were observational.
Blinding	Studies conducted on serum samples were observational and therefore not blinded. Blood obtained for the isolation of CD14+ monocytes was anonymised by the practitioner at the point of sample collection.

Reporting for specific materials, systems and methods

We require information from authors about some types of materials, experimental systems and methods used in many studies. Here, indicate whether each material, system or method listed is relevant to your study. If you are not sure if a list item applies to your research, read the appropriate section before selecting a response.

Materials & experimental systems

n/a	Involved in the study
☐	☒ Antibodies
☐	☒ Eukaryotic cell lines
☒	☐ Palaeontology and archaeology
☒	☐ Animals and other organisms
☒	☐ Clinical data
☒	☐ Dual use research of concern
☒	☐ Plants

Methods

n/a	Involved in the study
☒	☐ ChIP-seq
☐	☒ Flow cytometry
☒	☐ MRI-based neuroimaging

Antibodies

Antibodies used

All SARS-CoV-2 spike-specific monoclonal antibodies were produced in-house and have been previously described in Graham et al. Immunity, 2021. 54(6): p. 1276-1289 e6 and Seow et al. Cell Rep, 2022. 39(5): p.110757.

Primary antibodies expressed in-house:
CR3022 (Spike and RBD specific mAb); used at a range of concentrations.
CR3009 (N specific mAb); used at a final concentration of 2ug/ml for intracellular N staining.

Secondary antibodies:
Donkey anti-mouse IgG (H+L) highly cross-adsorbed secondary antibody, Alexa FluorTM 488 (Invitrogen #A21202).
Goat anti-mouse IgG (Fc-specific)-peroxidase antibody (Sigma #A2554).

Anti-Fc receptor antibodies:

For cell surface receptor expression determination by flow cytometry-
FITC anti-human CD64 antibody, clone 10.1, BioLegend #305006 (5ul per 10^6 cells)
FITC anti-human CD32 antibody, clone FUN-2, BioLegend #303204 (5ul per 10^6 cells)
FITC anti-human CD16 antibody, clone 3G8, BioLegend #302006 (5ul per 10^6 cells)

For blocking experiments -
Anti-human CD64 clone 10.1 mouse, BioLegend #305008 (3 ug/ml)
Anti-human CD32 clone IV.3 mouse, Caprico Biotechnology #102724 (3 ug/ml)
Anti-human CD16 clone 3G8 mouse, BioLegend #302008 (3 ug/ml)

Anti-IL-6 receptor antibody tocilizumab, MedChemExpress #HY-P9917 (20,100 and 500 ng/ml)

Validation

Commercial antibodies were validated by the manufacturers:
CD64 antibody, clone 10.1 https://www.biolegend.com/en-gb/products/apc-anti-human-cd64-antibody-5789
CD32 antibody, clone FUN-2 https://www.biolegend.com/en-us/search-results/apc-anti-human-cd32-antibody-662
CD32 antibody, clone IV.3 https://www.stemcell.com/products/anti-human-cd32-antibody-clone-iv-3.html
CD16 antibody, clone 3G8 https://www.biolegend.com/en-gb/products/purified-anti-human-cd16-antibody-571

CR3022 and murinized CR3009 antibodies were validated in Pickering et al PLoS Pathog. 2020 Sep 24;16(9):e1008817 and Graham et al. Immunity, 2021. 54(6): p. 1276-1289 e6.

SARS-CoV-2 spike-specific monoclonal antibodies were validated in Graham et al. Immunity, 2021. 54(6): p. 1276-1289 e6 and Seow et al. Cell Rep, 2022. 39(5): p.110757.

Eukaryotic cell lines

Policy information about cell lines and Sex and Gender in Research

Cell line source(s)	HEK293T/17 were obtained from ATCC (CRL 11268).<br>THP-1 were obtained from ATCC (TIB-202).<br>Vero-E6 were obtained from ATCC (CRL 1586) and modified to express TMPRSS2 as described previously (Winstone, H., et al., J Virol, 2021. 95(9)).<br>HeLa ACE2 were produced by Dr James Voss (Scripps Research, San Diego).<br>iPSC lines were provided by the HipSci Consortium and Prof. Fiona Watt.
Authentication	No authentication was performed.
Mycoplasma contamination	All cell lines tested negative for mycoplasma contamination.
Commonly misidentified lines (See ICLAC register)	No commonly misidentified cell lines were used.

Plants

Seed stocks	*Report on the source of all seed stocks or other plant material used. If applicable, state the seed stock centre and catalogue number. If plant specimens were collected from the field, describe the collection location, date and sampling procedures.*
Novel plant genotypes	*Describe the methods by which all novel plant genotypes were produced. This includes those generated by transgenic approaches, gene editing, chemical/radiation-based mutagenesis and hybridization. For transgenic lines, describe the transformation method, the number of independent lines analyzed and the generation upon which experiments were performed. For gene-edited lines, describe the editor used, the endogenous sequence targeted for editing, the targeting guide RNA sequence (if applicable) and how the editor was applied.*
Authentication	*Describe any authentication procedures for each seed stock used or novel genotype generated. Describe any experiments used to assess the effect of a mutation and, where applicable, how potential secondary effects (e.g. second site T-DNA insertions, mosiacism, off-target gene editing) were examined.*

Flow Cytometry

Plots

Confirm that:

- [x] The axis labels state the marker and fluorochrome used (e.g. CD4-FITC).
- [x] The axis scales are clearly visible. Include numbers along axes only for bottom left plot of group (a 'group' is an analysis of identical markers).
- [x] All plots are contour plots with outliers or pseudocolor plots.
- [x] A numerical value for number of cells or percentage (with statistics) is provided.

Methodology

Sample preparation	Flow cytometry assays for % SARS-CoV-2 infected cells were performed on THP-1 cell lines, therefore minimal sample preparation was required. Cells were fixed in 4% formaldehyde prior to intracellular staining for SARS-CoV-2 nucleocapsid.<br><br>For cell surface staining of primary monocyte-derived macrophages, peripheral blood mononuclear cells (PBMC) were separated from the peripheral blood of healthy donors by density-gradient centrifugation. PBMCs were washed twice with phosphate-buffered saline (PBS) supplemented with 2% FCS. Primary CD14+ monocytes were subsequently isolated by magnetic-activated cell sorting (MACS) with CD14+ Microbeads (Miltenyi Biotech, Bergisch Gladbach, Germany; #130-050-201) according to the manufacturer's protocol. CD14+ monocytes were washed, counted, adjusted to a density of 5 x 10^5 cells/ml and plated at 5 x 10^5 cells per well in 12-well plates, in the presence of RPMI supplemented with 10% FCS and 50 ng/ml human recombinant M-CSF (R&D Systems, UK, #216-MC-010/CF) for differentiation to macrophages for 4-5 days at 37°C with 5% CO2.<br><br>Macrophages were removed from plates using Accutase (Thermo Fisher Scientific, UK) then washed in FACS buffer. Blocking was performed with BD PharmingenTM Human BD Fc block (BD Biosciences #564220, UK; 5ul per 106 cells) for 15 minutes on ice before incubation with relevant antibody for a further 30 minutes on ice. Cells were washed twice in FACS buffer before flow cytometry.
Instrument	BD FACSCanto II.
Software	BD FACSDiva Software v9.2 was used to acquire data and FlowJo v10.8.1 (BD) for analysis.
Cell population abundance	N/A (THP-1 cell lines used or purified CD14+ monocyte-derived macrophages).

Gating strategy	For determination of % SARS-CoV-2 infected THP-1 cells by flow cytometry: debris was removed and main populations selected by plotting FSC-A vs SSC-A, and doublets were excluded by plotting FSC-H vs FSC-A and gating out cells that fell outside of the linear correlation. Negative controls (uninfected control cells, and uninfected control cells incubated with infection-mediating antibody, all permeabilised and stained with primary and secondary antibodies in parallel) were used to define boundaries for nucleocapsid-positive cells.<br><br>For determination of cell surface FcR expression on THP-1 cells or primary human macrophages by flow cytometry: debris was removed and main populations selected by plotting FSC-A vs SSC-A, and doublets were excluded by plotting FSC-H vs FSC-A and gating out cells that fell outside of the linear correlation. These populations were then used to determine expression of individual FcRs.

☒ Tick this box to confirm that a figure exemplifying the gating strategy is provided in the Supplementary Information.
